# Pre Dialysis care and types of Vascular Access Employed in Incident Hemodialysis Patients: A study from Pakistan

**DOI:** 10.12669/pjms.293.3385

**Published:** 2013

**Authors:** Sumbal Nasir Mahmood, Kunwer Naveed Mukhtar, Nousheen Iqbal, Syed Farrukh Umair

**Affiliations:** 1 Dr. Sumbal Nasir Mahmood, Diplomat American Board of Internal Medicine, Diplomat American Board of Nephrology, Department of Nephrology, Dr. Ziauddin University Hospital, Karachi**, **Pakistan.; 2 Dr. Kunwer Naveed Mukhtar, Diplomat American Board of Internal Medicine, Diplomat American Board of Nephrology, Department of Nephrology, Liaquat National Hospital & Medical College, Karachi, Pakistan.; 3 Dr. Nousheen Iqbal MBBS, FCPS I, Resident, Department of Internal Medicine, Dr. Ziauddin University Hospital, Karachi**, **Pakistan.; 4 Dr. Syed Farrukh Umair MBBS, FCPS I, Fellow, Department of Nephrology, Liaquat National Hospital & Medical College, Karachi, Pakistan.

**Keywords:** Hemodialysis, Arteriovenous fistula, Hemodialysis (HD) catheter, Vascular access, Patient referral, Predialysis nephrologic care

## Abstract

***Objective:*** To determine frequency of different vascular access use in Incident hemodialysis (HD) patients and determine whether predialysis care in terms of timely advice for vascular access placement was better in the hands of nephrologist.

***Methods:*** A cross sectional study was conducted. Data was collected on the type of access used for first HD, including temporary Central venous catheters (CVC), permanent CVC (Permacath), arteriovenous fistula (AVF), or arteriovenous graft (AVG). In addition, information was also gathered if patients were aware of their renal disease and was followed by other physicians or nephrologist.

***Results:*** A total of 120 patients were enrolled in the study, 80% required CVC as their first access for HD (96/120 patients) out of which 74.2% were dialyzed through temporary catheter and 5.8% through Permacath. About 20% of patients were dialyzed through mature Arteriovenous (AV) access. Majority (95.8%) of patients were being followed by any health care provider. 68% of them were aware of their renal disease. About 55.8% were referred to nephrologist and 40% were followed by other physicians. About 83.5% of patients followed by nephrologist were advised AV access prior to commencing HD, compared to only 10.4% followed by other physicians (p<0.05). 24/61 (39.3%) patients that were advised AV access by both groups had timely made AV access and underwent HD by it.

***Conclusion:*** Very high incidence of temporary HD catheter was used in Incident HD patients. Moreover, pre dialysis care in terms of placement of AV access prior to initiating HD is better in the hands of nephrologist and patients should be timely referred to nephrologist especially when they have Stage 4 chronic kidney disease (CKD).

## INTRODUCTION

An adequate vascular access for patients with End Stage Renal Disease (ESRD) on hemodialysis (HD) is one of the most important challenges that a nephrologist faces. Vascular accesses available for HD include arteriovenous fistula (AVF), arteriovenous graft (AVG), central venous catheters (CVC), both temporary and permanent (Permacath) that can provide adequate HD, however each of them have their limitations and advantages. Since native AVF is regarded as the best access for HD due to its high patency rate^1^^-^^3^ and lower mortality risk^4^^,^^5^ it is always considered as the first choice for HD patients, and is preferred over AVG and CVC. The Clinical Practice Guidelines for vascular access of National Kidney Foundation’s Dialysis Outcomes Quality Initiative (DOQI) recommend use of arteriovenous (AV) access for HD over CVC.^6^ The blood flow rates of AV access are higher compared to CVC^7^ and also there is lower risk of infection,^8^ thrombosis,^9^ and central venous stenosis.^10^

In addition, DOQI guidelines recommend use of AVF over AVG due to lower risk of complications.^6^ Since CVC are associated with higher complications including increased risk of cardiovascular complications^11^^-^^15^ and mortality,^16^ their use should be limited with timely referral to nephrologist and surgeon so that AVF can be created and enough time is available for it to mature, as late creation can also result in high failure rate.^17^ Enough evidence exists that late referral to a nephrologist of patients with chronic renal failure results in increased morbidity and mortality.^18^

In Pakistan, because of financial constraints temporary HD catheters are placed in patients awaiting AVF to mature and ESRD patients require sometimes two to three catheters before a permanent AV access is ready for use. These results in high mortality and morbidity with significant economic and logistic problems for both patients and their health care providers. Hence it is generally agreed that patients with chronic kidney disease (CKD) who are expected to begin dialysis should be referred beforehand for surgery to create a permanent AV access.^19^ Ideal time of this being when Creatinine clearance (Cr Cl) is < 25 ml/min^3^ so that enough time is allowed for fistula maturation.

We conducted this study in Pakistan to determine frequency of different vascular access use in Incident HD patients and determine whether predialysis care in terms of timely advice for vascular access placement was better in the hands of nephrologist.

## METHODOLOGY

This cross sectional study was conducted at Nephrology departments of Dr. Ziauddin University Hospital from January 2011 to December 2011 on all Incident HD patients who were defined as all patients starting HD and continued to require long term hemodialysis.

The study was approved by the ethical committee of the hospital. Informed consent was taken from the patients. Data was collected on the type of access used for first HD, including temporary CVC, Permacath, AVF, or AVG. In addition, information was also gathered if patients were aware of their renal disease and were followed by other physicians or nephrologist, which was defined as at least one outpatient visit with the respective physician prior to commencing HD. Timely advise for vascular access was considered if patients were informed that they will be needing dialysis therapy soon, and should have permanent vascular access placed prior to starting dialysis therapy. Inclusion Criteria included all patients who started HD for the first time and continued to require HD as a permanent means of renal replacement therapy as they were found to have ESRD. Exclusion criteria included all patients with acute renal failure, patients with acute or chronic renal failure that had CKD stage 3 three months prior to admission and patients already on HD that required a new access due to failed previous access.

***Statistical Analysis:*** Data was entered on SPSS version 14. Frequencies were calculated for gender, co-morbids, types of angioaccess use in first hemodialysis, follow up by other physicians and nephrologists and patient’s awareness of their renal disease. Mean and standard deviation was calculated for age. Chi square test of association was used to compare advice of AVF before initiating HD by other physicians with nephrologist. P value of < 0.05 was considered as significant.

## RESULTS

A total of 120 patients underwent HD for the first time in our hospital from January 2011 to December 2011. Their demographics are shown in Table-I. Most patients (80%) required CVC as their first access for HD (96/120 patients) out of which 74.2% were dialyzed through temporary catheter and 5.8% through permacath. About 20% of patients had a permanent, mature AV access that was used as their first access. Details are shown in Table-II.

**Table-I:** Demographic Characteristics of the Population.

*Age*	*54.9±15.2 yrs*
*Gender*	
Male Female	69 (57.5%) 51(42.5%)
*Co-Morbids*	
Type2 DM Hypertension Others	58 /120 (48.3%) 86/120 (71.1%) 30/120(25%)

**Table-II:** Angioaccess at the time of first Hemodialysis.

Temporary Catheter	89 (74.2%)
Permacath	7 (5.8%)
AV Fistula	22 (18.3%)
AV Graft	2 (1.7%)

**Table-III:** Frequency of Follow-ups.

Follow-up by other Physicians	48 /120 (40%)
Follow-up by Nephrologists	67 /120 (55.8%)
Follow –up by none	5/120 (4.2%)
Awareness about renal Disease	82 /120 (68%)

**Table-IV:** A/v fistula Advice by different health care provider.

*Primary Health care provider*	*AVF advised*		*Total*	*P Value*
	*Yes*	*No*		
Physicians	5(10.4%)	43(89.6%)	48	<0.001
Nephrologists	56(83.6%)	11(16.4%)	67	
First HD with AV access	24(20%)	96(80%)	120	

Data was also collected about frequency of patient follow up by various physicians and if they were aware of their renal disease status (Table-III). About 95.8% of patients were being followed by any health care provider out of which 68% were aware of their renal disease. Only 55.8% were referred to nephrologist, and 40% continued to be followed by other physicians till they required HD. About 4.2% of patients were never seen by any health care provider despite having risk factors of renal disease and uremic emergencies were their first presentation that required urgent HD.

A big proportion of patients 56/67 (83.5%) followed by nephrologist were advised AV access prior to commencing HD, compared to only 5/48(10.4%) followed by other physicians, a result that was statistically significant (p<0.05) (Table-IV) in terms of better predialysis care by nephrologist.

About 24/61 (39.3%) patients that were advised AV access by both groups had timely made AV access and underwent HD by it.

## DISCUSSION

Our study showed a very disturbing trend of late referral to nephrologist and hence a very high use of CVC especially temporary catheters and lower use of AVF in Incident HD patients. About 40% of patients with advanced renal failure were never referred to nephrologist till the time they required emergency HD. This trend is similar to that reported from Europe and United States where it is estimated that 20% to 57%^20^^, ^^21^ of patients are referred vey late and 66% of patients in the United States initiate maintenance HD therapy with a catheter.^21^ This trend was especially seen in patients followed by non nephrologist, as only 10% of the patients that were followed by these physicians were advised AV access prior to commencing HD despite having documented Stage IV CKD. These trends were seen after introduction of guidelines that strongly recommend AVF as the access of first choice.^22^^,^^23^

This also identifies that a big gap exists between guidelines and practice patterns. Our study also highlights the importance of timely referral to nephrologist as shown in various studies.^24^^-^^26^ Predialysis care was better in patients followed by nephrologist as most of them either had a functioning AV access or were advised AVF prior to starting HD and were aware of their renal status. This was also shown by Stehman Breen et al,^27^ who found that patients informed of their kidney disease more than one year before beginning dialysis therapy were nearly three times as likely to have a fistula placed than those informed one to four weeks before dialysis therapy. In the CHOICE study Astor et al^28^ also showed that patients referred to a nephrologist at least one month before long term HD therapy was initiated were more than three times as likely to have a fistula than a dialysis catheter as their first access.

HD was done through AV access only in a small number of patients(only 39.3%), even in patients that were aware of their renal disease and were advised AV access prior to starting HD. The reason was multifactorial including late referral for nephrological care, patient’s denial and resistance to accepting and participating in plans for renal replacement therapy it or lack of funding. This was similar to reasons that were reported in the 2005 Clinical Performance Measures Project.^29^ At the same time very few patients followed by other physicians were advised AVF compared to those followed by nephrologist, where most patients were not only aware of their renal functional status but were also advised timely to have a permanent AV access placed, results of which were statistically significant (p<.01). Hence, patients followed by other health care providers at advanced stages of renal failure had inadequate predialysis care and were put at stake for development of all complications associated with CVC use as about 80% of patients in our study required CVC as their first dialysis access. Similar results of use of CVC as initial access was shown by Medkouri et al^30^ in their study where 86.3% of patients initiated HD through a temporary catheter. Accumulating evidence suggests that the type of vascular access in use at initiation of HD is strongly related to future infectious complications, for example bacteremia^16^ central venous stenosis^4^^,^^12^ and mortality risk.^5^^,^^31^^,^^32^ This factor is modifiable and timely referral, implication and knowledge of guidelines need to be enforced to prevent morbidity.

Our results are similar to studies done in developed countries like United States and Canada, where CVC use as a first access is close to 60% and 70%^16^^,^^32^^,^^33^ respectively, however in these countries permacath is mostly used as CVC compared to our country where temporary HD catheters are placed and removed only if patient develops line sepsis or if catheter fails to function, resulting in increased cost as sometimes two to three catheters are placed while awaiting permanent AV access and also increased hospitalizations due to catheter related septicemia.

*Recommendations:* We recommend that patients should be timely referred to nephrologist especially when they have Stage 4 CKD. A close working relationship needs to be established between other physicians and nephrologists and surgeons so that timely placement of permanent AV access can be practiced. This will not only decrease morbidity and mortality risk in this susceptible patient population but also reduce the global cost of health care in ESRD patients. In addition, future studies are needed to look into factors as to why patients, even when advised AV access did not undergo dialysis through it, so that measures can be taken to reduce the incidence of temporary HD catheter use.

***Limitations of the study***: It was a cross sectional study and sample size was taken based on convenience sampling. Also follow up by health care providers was taken as only one outpatient visit prior to starting HD therapy, this might have influenced the results as physicians might not have enough time available to timely refer the patients for permanent AV access. However since the follow up was the same for both groups the statistical significance still holds.

## CONCLUSION

We report a very high incidence of temporary HD catheter use in Incident HD patients which is contrary to all the guidelines and recommendations of Nephrology societies. Also, pre dialysis care in terms of placement of AV access prior to initiating HD is better in the hands of nephrologist. Patient’s awareness of their disease and understanding is better with nephrologist.

## Authors Contribution

SNM: Is the main author, has taken responsibility of the integrity of the article as a whole, from its inception to its publication. KNM: Helped in composing the article, literature search and especially in writing down the discussion part of the Article. NI: She helped in the acquisition of data. SFU: He helped in with statistical analysis and writing of the article.
